# Cesarean section with operation on placental support as an adapted EXIT procedure for fetal thyroid goiter: A case report and literature review

**DOI:** 10.1002/ijgo.16060

**Published:** 2024-12-05

**Authors:** Sanpon Diawtipsukon, Woraluk Moradokkasem, Nareenun Chansriniyom, Pracha Nuntnarumit

**Affiliations:** ^1^ Department of Obstetrics and Gynaecology Faculty of Medicine Ramathibodi Hospital, Mahidol University Bangkok Thailand; ^2^ Department of Pediatrics Faculty of Medicine Ramathibodi Hospital, Mahidol University Bangkok Thailand

**Keywords:** adapted EXIT procedure, fetal thyroid goiter, OOPS procedure, operation on placental support, sustained placental cesarean section

## Abstract

The ex‐utero intrapartum treatment (EXIT) procedure is a specialized delivery strategy that extends utero–placental–fetal circulation to convert a potential neonatal emergency condition into a condition that is compatible with postnatal life. Cesarean section with operation on placental support is an EXIT technique that requires a relatively short duration of placental support and few skilled medical personnel and specialized instruments; it can successfully treat selected fetal indications. In the present study, we report a case of fetal thyroid goiter as an example of a fetal anomaly requiring the procedure. We then review all cases published in the medical literature that were similar to our procedure (15 cases including our new case). Twelve (80%) cases of fetal anomalies and three prophylactic cases of extremely low birth weight were delivered using a procedure adapted from the standard EXIT procedure. All 12 cases of fetal anomalies were treated by airway intervention. In 12 of the 15 cases (80%), direct laryngoscopy and endotracheal intubation were attempted; intubation was successful in seven of these 12 cases (58%). There was only one case of postpartum hemorrhage caused by uterine incisional bleeding, with a consequence of delayed hysterotomy closure. In summary, cesarean section with operation on placental support is a promising alternative delivery technique for neonates with airway obstructive lesions, especially in resource‐limited settings.

## Synopsis

Cesarean section with operation on placental support is an alternative delivery strategy for some fetuses with airway obstructive lesions, especially in resource‐limited settings.

## 1 INTRODUCTION

The ex‐utero intrapartum treatment (EXIT) procedure was first studied in non‐human primates in 1990^1^, ^2^, ^3^ and has been further developed to allow good perinatal outcomes in human fetuses who would be born with anomalies, especially lesions that might cause upper airway obstruction or other life‐threatening conditions requiring intervention immediately after birth.^4^, ^5^, ^6^ The procedure aims to maintain oxygenated utero–placental–fetal blood flow for as long as possible while the neonatal airway or cardiopulmonary function is suitably secured for postnatal life.^7^ Complexity levels for the procedure depend on neonatal patient requirements; a more complex intervention requires more time with placental support.^8^ The majority of EXIT procedures comprise EXIT‐to‐airway techniques in fetuses anticipated to have airway problems caused by conditions such as intrinsic defects in the larynx and trachea, congenital high airway obstruction syndrome (CHAOS), or oropharyngeal or cervical tumors compressing the airways.^9^, ^10^, ^11^ Fetal thyroid goiter is a common neck tumor causing fetal upper airway obstruction; it requires airway management in the intrapartum or immediate postpartum period.^12^, ^13^

The EXIT procedure is generally conducted by a skilled, multidisciplinary team comprising two maternal‐fetal medicine specialists/obstetricians who perform the cesarean operation, two anesthesia teams (anesthesiologists and nurse anesthetists) for both the pregnant woman and the afflicted neonate, one or more pediatric surgeons, one or more pediatric otolaryngologists, two surgical scrub and circulatory nurses, one or more neonatologists, and a pediatric nurse team for neonatal resuscitation. To maximize favorable neonatal outcomes and minimize maternal adverse events,^14^, ^15^, ^16^ specialized operative and monitoring instruments are required, such as uterine stapling devices to perform hysterotomy, a sterile ultrasonographic probe, and a pulse oximeter to monitor the partially delivered fetus .^17^, ^18^ These requirements mean that the complete EXIT procedure is difficult to perform in resource‐limited hospitals. However, the core concept of prolonged uteroplacental circulation by sustained placental support may be adapted to provide newborn airway protection under specific conditions.

There have been a few cases in which conceptual modifications of the EXIT procedure have allowed for intrapartum neonatal airway treatment. Herein, we report a favorable outcome using cesarean section (C‐section) with operation on placental support (OOPS) as an adapted EXIT procedure for a case of fetal thyroid goiter in a resource‐limited hospital setting. We also provide a short literature review of similar cases, focusing on prenatal diagnosis, C‐section technique, neonatal airway management, and neonatal and maternal outcomes.

## 2 CASE PRESENTATION

A 37‐year‐old woman, gravida 1, was admitted to the complicated obstetric ward for in‐patient fetal well‐being surveillance because of fetal hydrops. The patient had been diagnosed with Graves' disease 12 years before pregnancy. After multiple antithyroid drugs failed to normalize her thyroid function, radioactive iodine therapy was used as a definitive treatment. However, the patient developed post‐radioactive iodine hypothyroidism and was prescribed life‐long levothyroxine therapy. She received her first antenatal care at 7^0/7^ weeks of gestation (GA). At the antenatal care clinic, the maternal euthyroid clinical symptom was documented. A physical examination revealed unremarkable vital signs but was positive for thyroid‐associated ophthalmopathy, including lid lag, lid retraction, and exophthalmos in both eyes. The maternal thyroid gland was neither enlarged nor bruised. The antenatal laboratory examination appeared unremarkable. Maternal thyroid receptor antibody (TRAb) was concurrently checked with a thyroid function test; she had TRAb >40 IU/L, thyroid‐stimulating hormone 0.299 μIU/L, and free thyroxine 1.21 ng/dL. An ultrasound confirmed an intrauterine pregnancy and a viable fetus.

A routine fetal anatomic ultrasound scan at 19^6/7^ GA weeks demonstrated fetal tachycardia (175–185 beats/min [bpm]), fetal thyroid goiter (thyroid circumference 45.4 mm; 95th percentile [P_95_] = 33.0),^19^ and signs of fetal heart failure (mild cardiomegaly, tricuspid regurgitation, and pericardial effusion of 6 mm at the right ventricular wall),^20^ although the maternal clinical thyroid status was stable. Because of the abnormal prenatal ultrasound findings, fetal goitrous hyperthyroidism was considered.^21^, ^22^ Diagnostic cordocentesis was performed for a fetal thyroid function test; the results were thyroid‐stimulating hormone 0.015 μIU/L (P_5_–P_95_ = 1.2–8.4), free thyroxine 4.61 ng/mL (P_5_–P_95_ = 0.15–0.58), free triiodothyronine 9.67 pg/mL (P_5_–P_95_ = 0.13–0.33), total thyroxine 22.1 μg/dL (P_5_–P_95_ = 1.17–4.66), and total triiodothyronine 254 ng/dL (P_5_–P_95_ = 6.5–32.5).^23^ In fetal cord blood, TRAb 88.40 IU/L was documented. Amniotic fluid examination revealed a 46, XX karyotype and normal chromosomal microarray.

**FIGURE 1:**
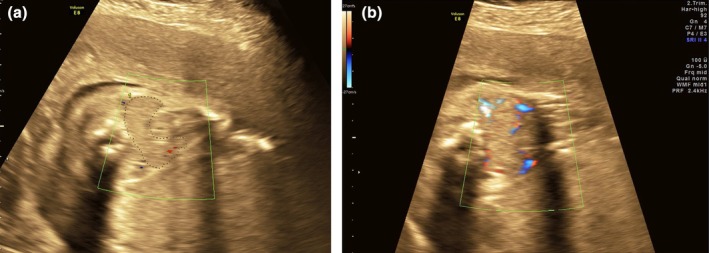
Prenatal ultrasound demonstrates fetal thyroid gland enlargement. (a) Fetal thyroid circumference. (b) Color Doppler ultrasound reveals peripheral vascular pattern fetal hyperthyroidism.

Although the maternal hypothyroidism was treated with levothyroxine, the fetal hyperthyroidism was treated with a transplacental antithyroid medication, methimazole, as a “block and replace” regimen. Maternal thyroid function was serially monitored and targeted as euthyroidism. Two weeks after the in utero fetal medical treatment, the fetal heart rate had declined to 140 bpm but the fetal thyroid gland remained enlarged. At 25^2/7^ GA weeks follow‐up ultrasound showed fetal hydrops consisting of skin edema at the scalp area, ascites, and increased pericardial effusion. Signs of fetal hyperthyroidism had also worsened, with an enlarged thyroid gland (Figure 1) of 7.85 mm in circumference (P_95_ = 4.1)^19^ and cardiomegaly with holosystolic tricuspid regurgitation. The patient was hospitalized for daily surveillance of fetal well‐being. A multidisciplinary team discussed intrapartum fetal airway management and planned for a C‐section with OOPS, adapted from the EXIT procedure.

On the basis of fetal hydrops progression and a non‐reassuring fetal status, a low transverse C‐section with OOPS was performed at 26^2/7^ GA weeks under deep general anesthesia. Standard maternal monitoring with an arterial line was conducted before rapid sequence induction with intravenous propofol and succinylcholine, followed by maternal endotracheal intubation. Inhaled desflurane and additional nitroglycerine were titrated to achieve deep anesthesia and uterine relaxation. Fetal anesthesia was received through the placental passage only. If a neonatal incision was required, a neonatal intramuscular cocktail of fentanyl, atropine, and vecuronium was planned. The intraoperative team comprised two obstetric surgeons, an operative scrub nurse, and a skilled neonatologist who was familiar with endotracheal intubation for extremely low‐birth‐weight newborns. The instruments for neonatal intubation, comprising a laryngoscope with appropriate blades, uncuffed endotracheal tubes of varied sizes, and guidewires, were prepared by the pediatric nurse on a separate sterile operative table.

**FIGURE 2:**
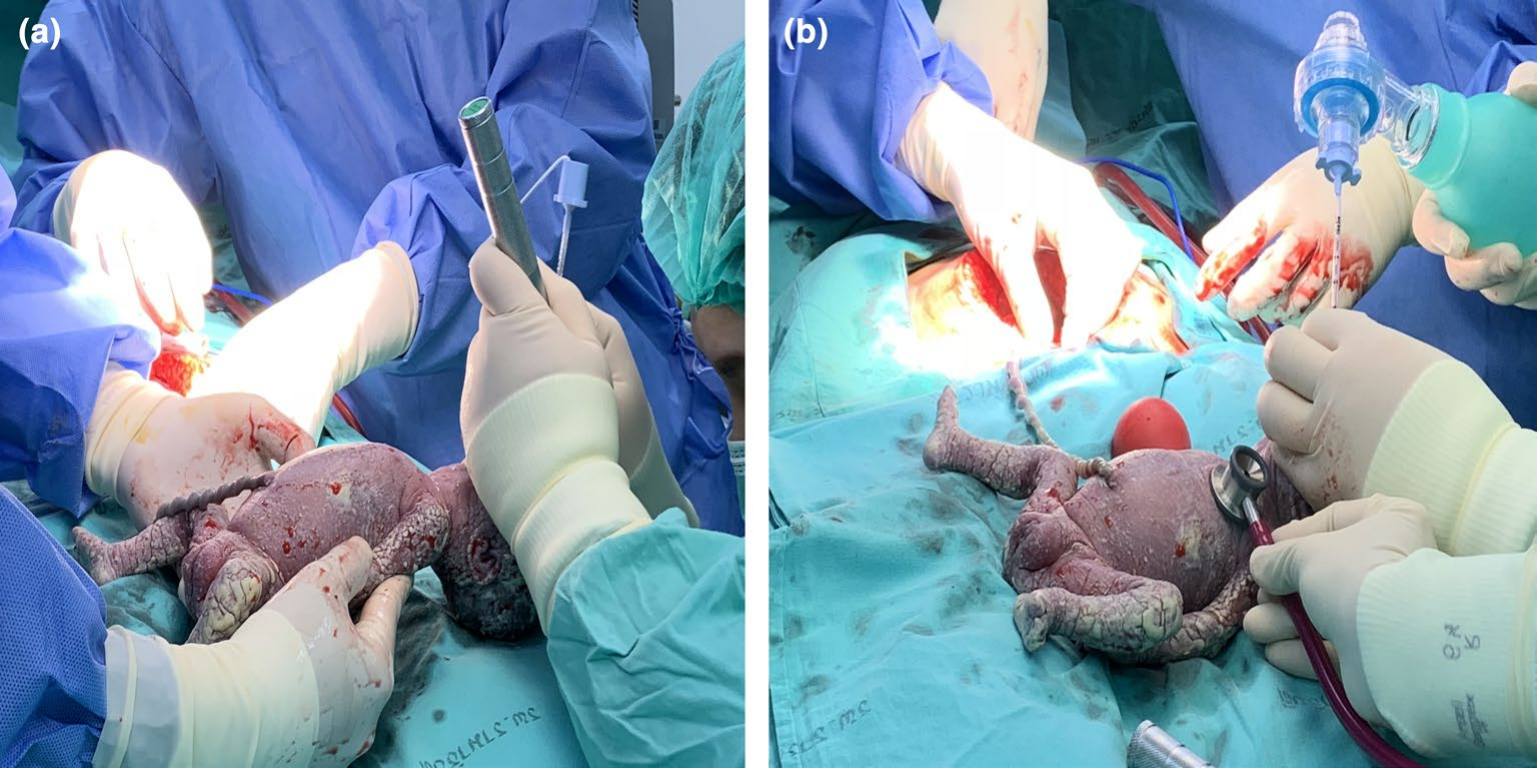
Successful endotracheal intubation by direct laryngoscope. (a) Performed by sterile intraoperative‐fielded neonatologist. (b) Umbilical cord pulse assessment by an obstetrician until the neonatal airway was secured.

After complete fetal delivery, the surgeon continuously checked umbilical cord pulsatility to ensure a sustained blood supply to the neonate, and uterotonic drugs were delayed to prevent placental separation. The intraoperative neonatal endotracheal intubation was planned to be abandoned if the neonatologist was unable to intubate within 15 min after birth, if no umbilical cord pulsation occurred, or if there was placental separation bleeding. The preterm female newborn was successfully endotracheally intubated using a No. 2.5 tube (depth 6.5 cm) at 2 min after birth, with stable maternal anesthetic status (Figure 2). Umbilical cord clamping and cutting were performed, and the intubated neonate was transferred to a second neonatal care team for further appropriate management. The placenta was delivered and vigorous oxytocin intravenous infusion was administered to stimulate uterine contraction and prevent postpartum hemorrhage. The maternal blood loss was estimated at 500 mL, with routine postoperative C‐section care.

**FIGURE 3:**
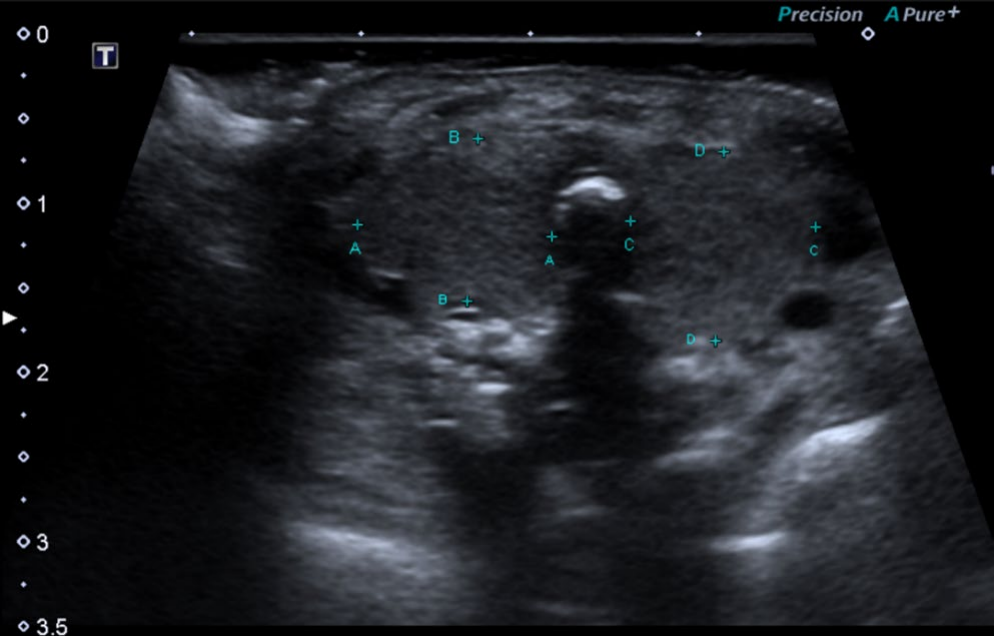
Postnatal thyroid gland ultrasound demonstrates diffuse enlargement.

The female newborn (weight 790 g, Apgar scores of 1, 6 T, and 6 T at 1, 5, and 10 min, respectively) was admitted to the neonatal intensive care unit with endotracheal ventilation (faction of inspired oxygen 0.5, peak inspiratory pressure/positive end‐expiratory pressure 29/7, oxygen saturation 95%). Postnatal physical examination revealed an enlarged thyroid gland, which was confirmed by ultrasound measurement (right lobe 225 × 102 × 142 mm, left lobe 195 × 121 × 132 mm; Figure 3). Neonatal thyroid function tests confirmed congenital hyperthyroidism; she was treated with methimazole, which was titrated until euthyroid status and discontinued within 1 month. The neonatal thyroid gland spontaneously reduced in size without any surgical intervention. Appropriate care for an extremely low‐birth‐weight preterm newborn was provided; she was extubated on day 6 of life and discharged after 4 months' hospitalization. No antithyroid drugs were provided at discharge, and monthly thyroid function tests were administered to ensure normal thyroid status.

This case report and literature review were approved by the Human Research Ethics Committee, Faculty of Medicine Ramathibodi Hospital, Mahidol University (COA no. MURA2024/366) and fully complied with international guidelines for human research. Written informed consent was obtained from the patient to publish this case report and the images shown.

## 3 LITERATURE REVIEW

A systematic search of the PubMed, National Center for Biotechnology Information, and MEDLINE databases was conducted to identify articles and reported cases involving modified EXIT procedures as a tool for intrapartum neonatal treatment. The authors combined the following keywords for original paper identification: “adapted EXIT,” “modified EXIT,” “delayed placental delivery,” “delayed cord clamping,” “sustained placental delivery,” “incomplete EXIT,” “unsuccessful EXIT,” and “intrapartum treatment or therapy.” Because few relevant publications were identified, the authors added “EXIT procedure” as a search term. Reports were carefully reviewed by two co‐authors to identify operations that were adapted or modified from the original EXIT procedure to provide total or complete fetal delivery before neonatal airway treatment, followed by umbilical cord clamp and placental delivery. Only English language articles were included, and irrelevant and duplicate articles were removed. Fifteen cases,^24^, ^25^, ^26^, ^27^, ^28^, ^29^, ^30^, ^31^, ^32^, ^33^, ^34^ including ours, were identified; their details are summarized in Table 1. Herein, we present a detailed analysis of the relevant information from these cases.

**TABLE 1:** Summary of the detail of each reported case in the literature.

First author (year)	Case	Prenatal diagnosis					Delivery					Outcomes		
		Week of diagnosis	Diagnosis US or MRI findings	Organ involvement	Hydramnios	Hydrops	Week of delivery	Time	Neonatal procedures	Time to secure (min)	Anes	Neonatal outcomes	EBL (mL)	PRC
Catalano et al. (1992)^24^	1	N/A	A large cystic‐solid epignathus teratoma 21 cm in length	Posterior nasopharynx mass exiting the mouth	Yes	No	37^0/7^	P	Tracheostomy	3	GA	Subtotal excision alive and well at 1 year	N/A	N/A
Skarsgard et al. (1996)^25^	2	23^0/7^	Cystic hygroma multicystic cervical mass 8.3 × 6.7 × 4.1 cm	Left anterior neck	No	No	30^0/7^	P	ET tube	6	GA	Subtotal excision DOL 2 discharge DOL 17	Minimal	N/A
Walker et al. (2005)^26^	3	35^0/7^	Teratoma 8 cm solid mass	Left anterolateral neck	Yes	No	35^2/7^	P	ET tube: failed rigid bronchoscope	5	GA	Excision DOL 1 healthy at 8‐year follow‐up	N/A	N/A
	4	N/A	A large cystic hygroma	Cervical and thoracic region	N/A	N/A	34^0/7^	E	ET tube: failed rigid bronchoscope	N/A	N/A	Extubated DOL 20 6 intralesional agent injections; excision at age 12 months	N/A	N/A
Costello et al. (2010)^27^	5	20^2/7^	Micrognathia	Mandible	Yes	No	37^1/7^	P	Direct laryngoscope: failed tracheostomy	N/A	N/A	Cornelia de Lange syndrome	N/A	N/A
Sumiyoshi et al. (2010)^28^	6	N/A	Teratoma 10 cm	Sphenoid bone with lesion extruding oral cavity and noses	N/A	N/A	28^5/7^	P	Tracheostomy	N/A	N/A	4 operative interval removals; Complete resection at age 6 months	N/A	N/A
Chung et al. (2012)^29^	7	20^4/7^	Epignathus teratoma mass 9.4 × 10.0 × 10.1 cm	Nasopharynx oral cavity	Yes	No	27^5/7^	E	Bronchoscope: failed tracheostomy	11	GA	Hospitalization 43 weeks PCA	2000	2 units
Pivetti et al. (2014)^30^	8	25^0/7^	CHAOS Expanded lungs Flat diaphragm	Larynx: subglottic membrane	N/A	Yes	32^1/7^	E	ET tube	3	SA	Mechanical ventilation 15 days Discharge in spontaneous breathing 38^1/7^ weeks PCA	N/A	N/A
Hung et al. (2017)^31^	9	26^2/7^	PPROM with chorioamnionitis ELBW neonate	–	No	No	26^2/7^	P	ET tube	N/A	EA	Smooth respiration at 1 month	N/A	N/A
	10	26^2/7^	PPROM with chorioamnionitis ELBW neonate	–	No	No	26^2/7^	P	ET tube	N/A	EA	Smooth respiration at 1 month	N/A	N/A
	11	26^2/7^	PPROM, ELBW neonate	–	No	No	26^4/7^	P	ET tube	N/A	N/A	Weaning tube at 1 month	N/A	N/A
Duek et al. (2018)^32^	12	32^2/7^	Pierre Robin fetus Severe retro‐micrognathia Dropped tongue, cleft palate	Mandible Oral cavity and pharynx	Yes	No	37^0/7^	P	Direct laryngoscope: failed Tracheostomy	N/A	N/A	Spontaneous breathing DOL 4 Healthy at 2‐year follow up	<1000	N/A
Kumar et al. (2019)^33^	13	33^0/7^	CHAOS Enlarge echogenic lungs, flat diaphragm, dilated trachea	Larynx: supraglottic atresia	N/A	No	38^0/7^	Vg	ET tube: failed Tracheostomy	6	No need	Alive at 1.5 months Surgery plan at age 8 months	No PPH	N/A
Yanko et al. (2023)^34^	14	20^6/7^	Nasopharyngeal teratoma 6 × 6 × 4 cm heterogenous mass	Anterior right nasal cavity	N/A	N/A	38^6/7^	P	ET tube	N/A	EA	Complete excision DOL 6	500	N/A
Diawtipsukon (current case)	15	19^6/7^	Thyroid goiter Circumference 78.5 mm	Midline anterior neck	No	Yes	26^2/7^	P	ET tube	2	GA	Extubated DOL 6 Discharge at age 4 months	500	No

Abbreviations: Anes, anesthesia technique; CHAOS, congenital high airway obstruction syndrome; DOL, day of life; E, emergency cesarean section; EA, epidural anesthesia; EBL, estimate blood loss; ELBW, extremely low‐birth‐weight, ET, endotracheal, GA, general anesthesia, N/A, not available; P, planned cesarean section; PCA, postconceptional age; PPH, postpartum hemorrhage; PPROM, prelabor premature rupture of membrane; PRC, packed red cells; SA, spinal anesthesia; Vg, vaginal delivery.

### 3.1 Prenatal diagnosis/indication for intrapartum treatment

Of the 15 cases involving intrapartum neonatal airway management, three were prophylactic cases of extremely low birth weight requiring resuscitation at birth, and 12 were cases of fetal anomalies (any pathology). Five of these 12 cases (42%) were diagnosed with teratoma with anticipated upper airway obstruction (one cervical teratoma case, four epignathus cases), and there were two cases each of cystic hygroma, micrognathia, and CHAOS. Only the current fetal thyroid goiter case involved protecting the neonatal airway at birth using the C‐section with OOPS procedure. Polyhydramnios was described in five cases, and only two cases (including ours) were diagnosed prenatally with hydrops.

### 3.2 Organ involvement

All of the 12 reviewed cases of fetal anomalies involved organs of the upper airway, which were treated by the adaptation or modification of the EXIT‐to‐airway procedure. Of the 10 cases with extrinsic causes of airway obstruction, 4 cases (40%) involved anterior neck masses compressing the fetal airway: two cystic hygroma cases, one cervical teratoma case, and the present case. The other cases comprised four cases of epignathus (an oropharyngeal teratoma that extrudes the oral or nasal cavities) and two severe cases of micrognathia (Cornelia de Lange and Pierre Robin syndromes). The other two cases involved pharyngeal stenosis (one membrane and one with atresia) and were prenatally diagnosed with CHAOS, which is classified as intrinsic airway obstruction.

### 3.3 Neonatal airway management

In 12/15 cases (80%), direct laryngoscopy was initially attempted to secure the neonatal airway by endotracheal intubation. Endotracheal intubation was achieved in 7/12 cases (58%). In the five cases in which endotracheal intubation was unsuccessful, treatment proceeded to rigid bronchoscopy in two cases and tracheostomy in three cases. Bronchoscopy was initially attempted in one case (case 6 in Table 1), but a tracheostomy was ultimately performed because of the neonate's small size and short umbilical cord. All three cases of large epignathus extruding from the oral and nasal cavities involved tracheostomy at birth. In 6/15 cases (40%), the neonatal airway was ultimately secured by tracheostomy. In 3/4 cases of teratoma (75%) a tracheostomy was required to secure the airway. The reports of only seven cases mentioned the time from neonatal birth to definite airway management (mean 5.1 min, range 2–11 min).

### 3.4 Anesthetic and surgical aspects

The reports of 10/15 cases (67%) provided details of the maternal anesthetic technique. Five cases (including the current case) received general anesthesia, three cases received epidural anesthesia, and one received spinal anesthesia. No anesthesia was necessary for one case (case 12 in Table 1), who delivered vaginally. Eleven of the 15 cases (73%) received planned C‐section deliveries, and 3 (20%) received emergency C‐section deliveries because of labor pain (two cases) or maternal worsening of Ballantyne's syndrome (one case). The final case had true labor pain but underwent successful vaginal delivery with neonatal intact cord resuscitation (Table 1). Only five cases reached term delivery (range 37^0/7^–38^6/7^ GA weeks); the other cases were dependent on the fetal condition for the time of delivery. Preterm labor (range 26^2/7^–34^0/7^ GA weeks) occurred in four cases.

### 3.5 Neonatal and maternal outcomes

There was no neonatal mortality in the 15 neonates who required an airway procedure at birth; the respiratory system was successfully secured in all cases using a range of different procedures. There was only one case (case 6 in Table 1) of postpartum hemorrhage, with an estimated blood loss of 2000 mL caused by uterine incisional bleeding, resulting in a delayed hysterotomy closure.

## 4 DISCUSSION

Fetal neck masses can lead to compromised airways, typically because they place pressure on the trachea or other upper respiratory tract organs. Fetal thyroid goiter, which is defined as abnormal enlargement of the fetal thyroid gland and signals fetal thyroid dysfunction, may cause airway obstruction.^19^, ^35^ Thyroid goiter is one of the prenatally diagnosed fetal neck lesions that may warrant a C‐section delivery with the EXIT‐to‐airway procedure^36^; however, recently reported data indicate that airway management usually only requires endotracheal intubation within a few minutes after delivery, with brief sustained placental support.^37^, ^38^ From our perspective, the standard EXIT procedure does not provide direct maternal benefits and may represent potential risks to the mother during both the surgery and subsequent pregnancies.^39^ Therefore, using the fully prepared EXIT procedure for fetal thyroid goiter may be considered as using “a sledgehammer to crack a nut,” especially compared with its use for other large fetal neck masses such as cervical teratoma, epignathus, or huge venolymphatic malformation mass—all of which are indications for EXIT‐to‐advanced airway management, EXIT‐to‐resection, or EXIT‐to‐extracorporeal membrane oxygenation.^40^, ^41^, ^42^

In general, an EXIT procedure requires a skilled, collaborative multidisciplinary team consisting of maternal‐fetal medicine specialists, anesthesiologists, pediatric otolaryngologists with neonatal airway management skills, and/or pediatric surgeons if EXIT‐to‐resection is desired.^13^ Depending on the neonatal intervention plan, team‐specific nurses and operating room personnel may also be required. Improving the maternal surgical approach is likely the most critical initiative for preventing maternal intrapartum hemorrhage in EXIT procedures that require a long incision‐to‐fetal operation duration. Intraoperative ultrasound guidance of the placental margin and an absorbable uterine stapling device for hysterotomy sealing can also decrease bleeding from the uterine incision site. Nonetheless, even high‐resourced hospitals report a median maternal blood loss of 800–1200 (500–2000) mL and a median blood transfusion requirement of 1.5–2.0 (1–4) units during the EXIT procedure.^43^.

As described in a previous report, Domínguez‐Moreno et al.^14^ observed no differences in maternal hemorrhage or median hemoglobin decrease in patients with median maternal and fetal EXIT procedure operation times of 60 (35–180) min and 8.5 (3–24) min, respectively, compared with cases of elective C‐section. We found similar results in the current literature review; patients who underwent an adapted EXIT procedure or C‐section with OOPS had no major procedure‐related maternal hemorrhage, with just one case of postpartum hemorrhage (estimated blood loss 2000 mL) caused by uterine incisional bleeding, resulting in delayed hysterotomy closure. Consequently, we suggest that resource‐limited hospitals should consider adapting the EXIT procedure for selected patients, to minimize the potential maternal risk during newborn interventions aiming to achieve postnatal survival.

The findings of our reported case and literature review suggest that three levels of consideration for intrapartum or immediate postpartum neonatal airway treatment can be categorized according to the anticipated fetal airway obstructive pathology and expected neonatal treatment procedure (detailed in Table 2). Level 1 is the highest fetal risk group for postnatal airway obstruction, and the standard EXIT procedure is indicated.

**TABLE 2:** Level of consideration for immediate neonatal intra‐ or postpartum treatment procedure.

Procedure	Suggestive indication	Multidisciplinary team	Detailed exploration	Special preparation
Ex‐utero intrapartum treatment “*EXIT procedure*.” Expected neonatal procedure duration: 45–150 min^44^	EXIT‐to‐airway Cervical teratoma or any teratoma etiology of the fetal neck lesions *Epignathus* *Oropharyngeal tumor* *Nasopharyngeal tumor* Compressive cervical masses Severe or syndromic micrognathia^36^, ^45^ CHAOS Reversal of tracheal occlusion following a FETO procedure EXIT‐to‐surgery EXIT‐to‐ECMO	Sterile operative field MFM specialists/obstetricians Neonatologist(s) Neonatal anesthesiologist Pediatric otolaryngologist Scrub nurses (maternal and neonatal team) Pediatric surgeon*/pediatric CVT surgeon* *Depends on the pathology Attendance area Maternal anesthesiologist Nurse anesthetist (s) (maternal and neonatal team) Neonatal nurse team Operating room personnel	Anesthetic technique Deep general anesthesia Maintain uterine relaxation Stabilize maternal hemodynamics Restore uterine tone after cord clamp Additional fetal anesthesia Maternal aspect Uterine incision away from the placental margin Sealing of hysterotomy edge by stapling device Fetal aspect Gently partial delivery of fetal head and shoulder(s) Monitor fetal well‐being Neonatal procedure(s) are begun by surgeon(s) as an organized plan	Blood reservation availability Maternal hemodynamic monitoring Intraoperative sterile ultrasound for mapping Uterine stapling device Amnioinfusion to maintain uterine volume Intraoperative sterile ultrasound and pulse oximetry Neonatal treatment instruments such as ET tubes, laryngoscopes, rigid or flexible bronchoscope
Cesarean section with operation on placental support procedure “*C‐section with OOPS procedure*” Expected neonatal procedure duration: 5–20 min^46^	Thyroid goiter Cystic cervical tumor (any size) Vascular anomalies (both vascular tumors and malformations) *Lymphangioma* *Hemangioma* Complete vascular ring lesion *Double aortic arch* *Right aortic arch with left ductal arch* Pulmonary artery sling^a^	Sterile operative field MFM specialists/obstetricians Neonatologist(s) Scrub nurses (maternal and neonatal team) Pediatric otolaryngologist* *Depends on the pathology Attendance area Maternal anesthesiologist Nurse anesthetist (only maternal team) Neonatal nurse team Operating room personnel	Anesthetic technique Deep general anesthesia Maintain uterine relaxation Stabilize maternal hemodynamics Restore uterine tone after cord clamp Maternal aspect Low transverse uterine incision as usual Fetal aspect Complete fetal delivery Place perpendicular to the mother at the approximated level of the placenta MFM specialists monitor umbilical cord pulse Neonatologist(s) or pediatric otolaryngologist perform endotracheal intubation	Blood reservation availability Maternal hemodynamic monitoring Sterile neonatal treatment instruments: ET tube, laryngoscope
Procedure requiring a second team in the operating room “*PRESTO procedure*” OR Attended delivery procedure^8^	Non‐severe micrognathia^47^ Cleft lip and cleft palate Partial vascular ring *Aberrant right subclavian artery* Extremely low birthweight newborns Hydrocephalus or CNS anomalies considered for bilateral vocal cord paralysis	Sterile operative or delivery field MFM specialists/obstetricians Scrub nurses (only maternal team) Attendance area Neonatologist Neonatal nurse team Operating room personnel Anesthesiologist* Nurse anesthetist* (*only maternal team*) Pediatric surgeon* or otolaryngologist* outside the operative field *If needed	Anesthetic technique As usual as a standard delivery case Stabilize maternal hemodynamics Maternal aspect Cesarean section or vaginal delivery (less recommended) Fetal aspect Complete fetal delivery as usual Basic resuscitation by a neonatologist Neonatologist activates the neonatal procedure if needed	Regular blood reservation as a delivery case Maternal hemodynamic monitoring Basic neonatal resuscitation instruments Neonatal treatment instruments such as ET tubes, laryngoscopes, rigid or flexible bronchoscopes stand by in place

Abbreviations: CHAOS, congenital high airway obstruction syndrome; CVT, cardiovascular and thoracic surgeon; ECMO, extracorporeal membrane oxygenation; ET, endotracheal tube; FETO, fetoscopic endoluminal tracheal occlusion; MFM, maternal‐fetal medicine.

^a^ Pulmonary artery sling is categorized as a partial vascular ring lesion but compresses to the trachea posteriorly, and symptoms can be similar.^48^, ^49^

**FIGURE 4:**
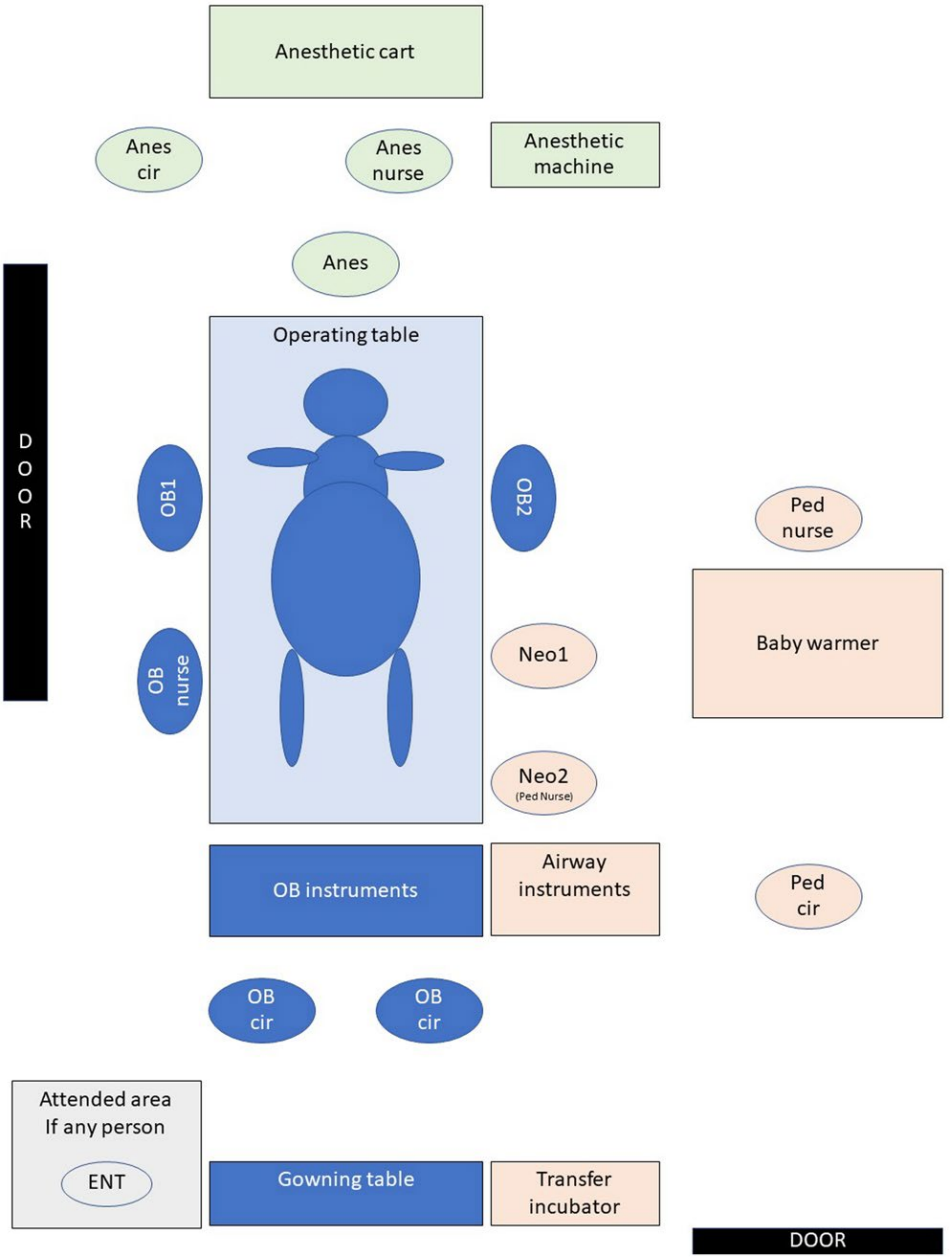
Operating room layout for a multidisciplinary team in cesarean section with operation on placental support (OOPS) procedure. Anes, anesthesiologist; Anes cir, anesthetic circulating person; Anes nurse, nurse anesthetist; ENT, attended otolaryngologist; Neo, neonatologist; OB, obstetrician; Ped cir, pediatric circulating person; Ped nurse, pediatric nurse.

In the second level (with an operating room layout as shown in Figure 4), C‐section with OOPS is indicated as an adapted EXIT procedure for fetuses who are prenatally diagnosed with a moderate risk of airway obstructive lesions, such as vascular anomalies of the neck or fetal thyroid goiter,^36^ similar to the current and other reported cases.^37^ Because vascular anomalies and some neck lesions are soft and grow around the airway rather than displacing it, most patients can be intubated through a laryngoscope at birth.^50^, ^51^ As noted in a previous study of fetal neck masses,^52^ mass location and size are not significantly associated with the incidence of airway intervention; however, nearly 100% of cervical lymphatic malformations over 3.5 cm in size are reportedly intubated at birth without using EXIT procedures. We therefore recommend that vascular anomalies of the neck should be taken as an indication that the neonatal airway requires a C‐section with OOPS. Notably, in a previous case series,^53^ “standard EXIT” was used to describe the perinatal management of compromised fetal airway lesions; however, the operating procedure details indicated that some babies were completely delivered from the uterine cavity and placed on an adjacent sterile field, similar to our C‐section with OOPS procedure.

The last level represents an alternative approach to neonatal airway management with a low potential for airway obstruction at birth: a PRESTO (procedure requiring a second team in the operating room)^47^ or an attended delivery procedure, which is also recommended by the International Pediatric Otolaryngology Group. However, universal positive outcomes cannot be expected.^8^, ^54^

To our knowledge, no previous studies or systematic reviews have examined an immediate post‐completed neonatal delivery airway treatment procedure during ongoing placental support or before umbilical cord clamping for fetal thyroid goiter; ours is therefore the first such report. Our study's major limitation involves the paucity of clinically relevant evidence to support the C‐section with OOPS procedure. Further prospective studies are required to support it, especially in terms of indications, patient counseling, and perinatal outcomes. Compared with the standard EXIT procedure, C‐section with OOPS has fewer demands in terms of human resources and specialized medical instruments; nonetheless, there are some maternal risks, including procedure‐related hemorrhage. Even in highly experienced centers,^55^, ^56^ use of the standard EXIT procedure has been decreasing because of advancements in imaging modalities to detail fetal airway anatomy^57^ as well as developments in instruments for neonatal airway management, which make successful intubation easier. Nonetheless, more cases are required to enable direct comparisons between the C‐section with OOPS and standard EXIT procedures in terms of their impact on neonatal and maternal outcomes and their costs. Regarding patient benefits, the present report is an example of a low‐resource setting that encourages the C‐section with OOPS procedure for specific newborn anomalies. However, there remains a need for standardized protocols for the C‐section with OOPS procedure.

## 5 CONCLUSION

The C‐section with OOPS procedure is a promising alternative delivery technique that provides prolonged placental support for neonates with specific airway obstructive lesions. Fetal thyroid goiter, cystic cervical tumors, and vascular anomalies may be considered an indication for this delivery strategy with immediate postpartum neonatal airway treatment, especially in settings in which the standard EXIT procedure cannot be used.

## AUTHOR CONTRIBUTIONS

All authors made significant contributions to this paper. S.D. conceptualized the study, managed patient care, collected data, reviewed the literature, and prepared the manuscript. W.M. and N.C. managed patient care and reviewed the relevant information in the related literature. P.N. organized the patient care team and critically revised the manuscript.

## FUNDING INFORMATION

Open‐access publication was provided by Mahidol University.

## CONFLICT OF INTEREST STATEMENT

The authors have no conflicts of interest.

## Data Availability

All data generated or analyzed during this study are included in this article. Further inquiries can be directed to the corresponding author.
